# Resolving Magnetopause Shadowing Using Multimission Measurements of Phase Space Density

**DOI:** 10.1029/2021JA029298

**Published:** 2022-01-27

**Authors:** F. A. Staples, A. Kellerman, K. R. Murphy, I. J. Rae, J. K. Sandhu, C. Forsyth

**Affiliations:** ^1^ Mullard Space Science Laboratory University College London London UK; ^2^ Department of Earth, Planetary, and Space Sciences University of California Los Angeles CA USA; ^3^ Northumbria University Newcastle upon Tyne UK

**Keywords:** radiation belt, electron dropout, magnetopause shadowing, geomagnetic storm, electron loss, PSD

## Abstract

Loss mechanisms act independently or in unison to drive rapid loss of electrons in the radiation belts. Electrons may be lost by precipitation into the Earth's atmosphere, or through the magnetopause into interplanetary space—a process known as magnetopause shadowing. While magnetopause shadowing is known to produce dropouts in electron flux, it is unclear if shadowing continues to remove particles in tandem with electron acceleration processes, limiting the overall flux increase. We investigated the contribution of shadowing to overall radiation belt fluxes throughout a geomagnetic storm starting on the 7 September 2017. We use new, multimission phase space density calculations to decipher electron dynamics during each storm phase and identify features of magnetopause shadowing during both the net‐loss and the net‐acceleration storm phases on sub‐hour time scales. We also highlight two distinct types of shadowing; “direct,” where electrons are lost as their orbit intersects the magnetopause, and “indirect,” where electrons are lost through ULF wave driven radial transport toward the magnetopause boundary.

## Key Points

- Multimission phase space density observations are necessary to resolve relativistic electron dynamics during September 2017 storm
- Relativistic electron losses to the magnetopause were identified, which led to further diffusion of electrons toward the magnetopause
- Electron loss to the magnetopause was observed simultaneous to prompt local energization in the heart of the radiation belt

## 1 Introduction

The outer radiation belt is a ring of relativistic electrons which are trapped by Earth's magnetosphere, surrounding the Earth at distances from ∼3 to 8 Earth Radii above Earth's surface. This population of particles has a range of energies between hundreds of keV and tens of MeV (Mauk et al., 2014), which can pose a hazard to the operation of satellites which lie within the belt (e.g., Baker, Kanekal, et al., 1994; Wrenn, 1995; Wrenn et al., 2002). Most of the time, the outer belt is slowly varying, however, during geomagnetic storms the particle flux in the outer belt by change by orders of magnitude in a matter of hours to days (e.g., Baker, Blake, et al., 1994; Morley, Friedel, Cayton, & Noveroske, 2010). Furthermore, a geomagnetic storm may result in a net‐increase, decrease, or no response in radiation belt flux (Reeves et al., 2003). Continuously competing acceleration and loss mechanisms, which act to create or remove relativistic electrons from the magnetosphere, determine the net flux of electrons in the outer belt.

Acceleration of electrons can occur via gyro‐resonant wave‐particle interactions between hundreds of keV “seed” electrons and very low frequency (VLF) whistler mode waves (Horne & Thorne, 1998; Horne et al., 2005; Meredith et al., 2002; Summers et al., 1998). Particles may also be energized by drift‐resonant wave‐particle interactions with ultralow frequency (ULF) waves (e.g., Elkington et al., 1999; Kellogg, 1959; Lejosne & Kollmann, 2020; Mann et al., 2013), or by ULF driven inwards radial diffusion (e.g., Fälthammar, 1965; Jaynes et al., 2015). This inward diffusion of electrons results in betatron acceleration which energizes electrons. Similarly, electrons are decelerated if they are transported outwards via radial diffusion. If the ring current is enhanced, electrons may also be adiabatically transported outwards, and decelerated (Dessler & Karplus, 1961; McIlwain, 1966).

Loss mechanisms act independently or in unison to remove electrons from the radiation belts. Electrons are lost by precipitation into the atmosphere, induced by wave‐particle interactions resulting in pitch‐angle scattering (e.g., Miyoshi et al., 2008; Rae et al., 2018; Rodger et al., 2015; Thorne & Kennel, 1971), by deceleration following outwards radial diffusion, or through the magnetopause into interplanetary space. The latter process is called magnetopause shadowing, and electrons are either lost directly to the compressed magnetopause intersecting drift paths (direct shadowing; Green et al., 2004; Kim et al., 2008; Li et al., 1997; Saito et al., 2010) or indirectly if electrons are transported toward the magnetopause boundary and subsequently lost (Loto'aniu et al., 2010; Morley, Friedel, Spanswick, et al., 2010; Rodger et al., 2019; Shprits et al., 2006). Direct shadowing is entirely controlled by the motion of the magnetopause, whereas indirect shadowing also depends upon the rate of outward radial diffusion, which is coupled to PSD gradients created by initial loss to the magnetopause and ULF wave power. As such, loss time scales for either mechanism will differ. While direct and indirect magnetopause shadowing both ultimately describe loss through the magnetopause, this study considers these processes separately to convey nuance in the physical mechanisms contributing to magnetopause losses.

Geomagnetic storms produce highly variable electron fluxes because both acceleration and loss mechanisms are enhanced, acting in separate locations and on a variety of time scales, from hours (Chaston et al., 2017; Mann & Ozeke, 2016) to several days over the duration of a storm (Forsyth et al., 2016; Murphy et al., 2018). It is understood that the radiation belts have a two‐step response to geomagnetic storms; a net‐loss phase when the radiation belt flux decreases overall during storm onset, followed by a net‐acceleration phase where radiation flux increases overall (Murphy et al., 2018). Electrons are usually lost from the belts before lower energy electrons are accelerated because of the way in which the magnetosphere responds to solar wind structures which drive geomagnetic storms. At storm onset, the magnetopause is often compressed by shock structures in the solar wind (e.g., Dmitriev et al., 2014; Sibeck et al., 1989), resulting in some loss of electrons via magnetopause shadowing. If the compression is large enough, losses due to shadowing are extreme, and the entire outer radiation belt may suddenly decrease by orders of magnitude over a time scale of hours; this is referred to as a dropout (e.g., Borovsky & Denton, 2009; Onsager et al., 2002). Dropouts are important precursors to the latter net‐acceleration phase as they remove both the existing radiation electrons and seed electrons, which limits the number of particles accelerated from this lower energy population (Bingham et al., 2018). The resulting net‐loss period may extend into main phase of the geomagnetic storm, then acceleration mechanisms begin to increase the overall electron flux. The net‐acceleration phase is delayed compared to the net‐loss phase because the time scales of wave driven acceleration and/or ULF driven diffusion are slower (hours to days; Baker, Blake, et al., 1994; Boyd et al., 2014; Elkington et al., 2003; Horne et al., 2005; Reeves et al., 2013). It is not known whether magnetopause shadowing continues to contribute to the overall flux during the net‐acceleration phase.

Due to the inherent rapid nature of shadowing, it is extremely hard to study the characteristics of dropout events in detail. So far, magnetopause shadowing observations include identifications of simultaneously compressed magnetopause and a net‐decrease in electron flux (e.g., Morley, Friedel, Spanswick, et al., 2010; Rodger et al., 2019) and/or measurements of butterfly pitch‐angle distributions near the compressed magnetopause (e.g., Kang et al., 2018; Ozeke et al., 2020; Tu et al., 2019). Such pitch‐angle distributions are an observational feature of shadowing since equatorial bouncing particles (with a high pitch angle) drift to higher radial distances at the magnetopause nose than high latitude bouncing particles (with low pitch angles), therefore high pitch‐angle electrons are preferentially lost to the magnetopause (Sibeck et al., 1987).

Magnetopause shadowing also shows specific characteristics identifiable in phase space density (PSD; Loto'aniu et al., 2010; Shprits et al., 2006; Turner et al., 2012). The collective dynamics of the radiation belts are often determined by using measurements of PSD, transformed into adiabatic invariant space *μ*, *K*, and L*. Radiation belt studies have notably used PSD to distinguish localized internal sources of high energy electrons (e.g., Boyd et al., 2014; Chen et al., 2006; Green & Kivelson, 2004; Miyoshi et al., 2003; Selesnick & Blake, 2000) from radial diffusion of an external source (e.g., Degeling et al., 2008; Jaynes et al., 2018; Ozeke et al., 2019).

Magnetopause shadowing is discernible in adiabatic coordinates through several features (illustrated in Figure 3 of Turner et al., 2012). There is an initial “high pressure” phase during storm onset where the outer boundary of the radiation belt is compressed such that a significant amount of the PSD distribution is lost to interplanetary space via direct magnetopause shadowing. When the pressure relaxes during the main storm phase, the magnetopause expands and there is a peak in PSD at the minimum radial distance which the magnetopause reached during the compression, and a strong negative gradient in PSD toward the expanded magnetopause. Over time, ULF wave activity during the main phase of the storm will rapidly diffuse the remaining particles down any radial gradients in the PSD profile, resulting in a decrease in the L* location of peak PSD, and a decrease in PSD at all L* compared to the prestorm distribution. Furthermore, combined negative phase space density gradient and enhanced ULF wave activity will lead to indirect magnetopause shadowing (Loto'aniu et al., 2010; Shprits et al., 2006; Turner et al., 2012).

While PSD has been used to analyze electron dropouts (e.g., Ma et al., 2020; Shprits et al., 2012; Turner et al., 2013; Xiang et al., 2017; Zou et al., 2020), the rapid time scales of electron loss via magnetopause shadowing have not yet be accurately characterized. Turner et al. (2014) made significant progress characterizing the time scales of dropouts but found that there was insufficient data to resolve an accurate time scale at all L*. This is because it is difficult to measure PSD profiles in L* on the time scales necessary with single or even dual spacecraft in geostationary transfer orbits. In this study, we investigate the dynamics of PSD during the early September 2017 geomagnetic storm using a new multispacecraft data set of PSD measurements that covers L* = 1.5–9. By using a multisatellite data set, we can measure variations in PSD at unprecedented temporal and spatial resolution to investigate the time scales of direct, and indirect, magnetopause shadowing.

## 2 Data

### 2.1 Phase Space Density Measurements

In this paper, we primarily use PSD to characterize the radiation belt response to a geomagnetic storm from 6 to 10 September 2017. PSD describes the kinematical state of radiation belt electrons using three coordinates of position, and the three components of canonical momentum (Schulz & Lanzerotti, 1974). Since the number of electrons in the radiation belt is sufficiently large and energetic, we can describe gyration, bounce, and drift motions in terms of three adiabatic invariants of motion, namely *μ*, *K*, and L* (Roederer, 1967). In this formalism, we consider only the phase‐averaged motion of particles, reducing the problem to a three‐dimensional approximation of the system. We then consider the density of electrons in this phase space, the PSD.

The benefit of using an invariant PSD is that nonadiabatic processes are easily identified by any changes to the PSD distribution; i.e., the PSD distribution does not change if only adiabatic changes are occurring to the system (following Liouville's theorem). Furthermore, the PSD distribution in L* is an important characteristic when interpreting indirect magnetopause shadowing processes because the PSD gradient in L* determines the rate of particle diffusion toward this outer boundary.

The electron PSD is here defined in units of (c/cm/MeV)^3^. For each spacecraft and instrument, the adiabatic invariants *μ*, *K*, and L* (Roederer, 1967) are computed using the International Radiation Belt Models (IRBEM) library (Boscher et al., 2013), the International Geomagnetic Reference Field (IGRF) internal field model, and the semiempirical Tsyganenko 2001 storm (T01s) external magnetic field model (Tsyganenko et al., 2003). Through the September 2017 storm, we use PSD measurements from

1. Van Allen Probes MagEIS (Magnetic Electron Ion Spectrometer) and REPT (Relativistic Electron‐Proton Telescope) instruments (Baker et al., 2014; Blake et al., 2014; Mauk et al., 2014)
2. THEMIS ESA (Electrostatic Analyzer) and SST (Solid State Telescope) instruments (Angelopoulos, 2008; Angelopoulos et al., 2008; McFadden et al., 2008)
3. MMS FEEPS (Fly's Eye Electron Proton Spectrometer) instrument (Blake et al., 2016; Burch et al., 2016)
4. GOES MAGED (Magnetospheric Electron Detector; Rodriguez, 2014a; Sillanpää et al., 2017) and EPEAD (Energetic Proton, Electron, and Alpha Detector; Rodriguez, 2014b)
5. GPS (Global Positioning System) Navstar Satellite CXD (combined X‐ray dosimeter; Tuszewski et al., 2004)

These PSD observations comprise of measurements from 32 satellites. A novel method is employed to remove identified statistical systematic bias, and to define the error in each observation. Using pairs of spacecraft, one spacecraft and instrument is chosen as a “gold standard,” and the correction is performed for each fixed energy channel on the other spacecraft. Conjunctions in phase space are found within 10 min, and 0.1 in L*, for fixed values of the three adiabatic invariants, and for all conditions and times. For this study, Van Allen Probe B and bias‐corrected GOES 15 data are used as gold standards to calibrate all the other data.

The bias and error corrections are specific to a particular energy channel, and to bins of the magnitude of the PSD, but not to any specific period. Distributions of the change in PSD for each bin are analyzed to determine the percentiles 5 through 95. The 50th percentile (median) represents the bias at a given PSD magnitude, while the interquartile range describes the error in the distribution. A numerical search for the best function which describes the bias offsets is conducted numerically, including exponential, power law, and polynomial functions; with and without *y*‐intercept offsets. The function that provides a fit with the lowest sum of absolute deviation over all the binned median values is chosen as the preferred solution for a given pair of spacecraft, instruments, and specific energy channel. The correction process is repeated for every energy channel, instrument, and spacecraft.

For this analysis, a sine pitch‐angle distribution is assumed for the GPS data; the pitch‐angle distribution obtained from the upper energy channel on MAGED is employed for the EPEAD observations; and THEMIS data are only included above *L* = 6 due to contamination within the radiation belts.

**Figure 1:**
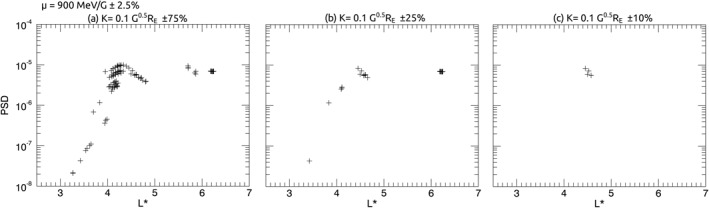
Intercalibrated phase space density (PSD) data over a 1‐hr time interval, plotted as a function of L* for *μ* = 900 MeV/G ± 2.5% and *K* = 0.1 G^0.5^ *R*_E_ within a range (a) *K* ± 75%, (b) *K* ± 25%, (c) *K* ± 10%.

Rather than interpolating PSD observations to find PSD for specific *μ* and *K*, as is traditionally practiced, in this study, we use all PSD measurements which lie within a specified range of *μ* and *K* to represent a specific population of electrons. Ranges of *μ* and *K* are chosen by visually inspecting profiles of PSD across L* for a variety of data ranges to find the range which (a) provides PSD profiles with sufficient data across L* and (b) does not present PSD with multiple distinct characteristics which are dependent upon *K*. A small *μ* range was chosen to minimize overlap between measurements taken by different energy channel ranges for different satellites, while a relatively large range of *K* was chosen to maximize PSD data availability across all L*. Though we note that variability of *K* introduces some variability in energy as smaller pitch angles will require a larger momentum for the same value of *μ*. Figure 1 shows a comparison of different ranges of *K* to demonstrate how data ranges are chosen, where Figure 1b is the optimal data range of *K*.

For *μ*, we use a range of ±2.5%; e.g., for a specified *μ* of 400 MeV/G, the range is 390–410 MeV/G. The range of *K* used is ±25%; e.g., for a specified *K* 0.1 G^0.5^ *R*_E_, the range is 0.075–0.125 G^0.5^ *R*_E_. This approximately corresponds to equatorial pitch angles between ∼40° and 70° during the prestorm period, or between ∼30° and 50° at minimum SYM‐H.

### 2.2 Parameterization of the Outer Boundary

In this study, we use both observations of the magnetopause and calculations of the last closed drift shell (LCDS) when considering the outer boundary of the radiation belts. We calculate the LCDS numerically as the maximum L* at which an electron with a constant *K* follows a closed drift path in the T01s external magnetic field model, where there is a single magnetic minimum along a field line to exclude bifurcated drift shells.

The magnetopause location is represented by the Shue et al. (1998) magnetopause model, and direct observations of magnetopause crossings by the THEMIS and GOES spacecraft, as recommended by Staples et al. (2020). To identify THEMIS magnetopause crossings, magnetic and plasma measurements are taken from the Fluxgate magnetometer (Auster et al., 2008) and the Electrostatic Analyzer (McFadden et al., 2008). For GOES 13 and 15 magnetopause crossings, magnetic field data from the flux gate magnetometers were used (Singer et al., 1996). The geometric shape of the Shue et al. (1998) magnetopause model is used to estimate the distance of the magnetopause at the subsolar point for a spacecraft magnetopause crossing at any solar‐zenith angle (e.g., Staples et al., 2020), the “equivalent” subsolar standoff distance.

### 2.3 Solar Wind Data

Solar wind data and geomagnetic indices used are provided by the NASA/Goddard Space Flight Centers OMNI data set through Coordinated Data Analysis Web (CDAWeb; https://omniweb.gsfc.nasa.gov/). Solar wind measurements in this data set are taken by the ACE, Wind, IMP 8, and Geotail missions, and are propagated to the bow shock nose. The SYM‐H index, calculated in a similar manner to Dst index by ground‐based midlatitude magnetometer stations, is used to indicate geomagnetic activity (Iyemori, 1990; Iyemori et al., 2010; Wanliss & Showalter, 2006). All data used from CDAWeb have 5‐min resolution.

## 3 Case Study Overview: September 2017

**Figure 2:**
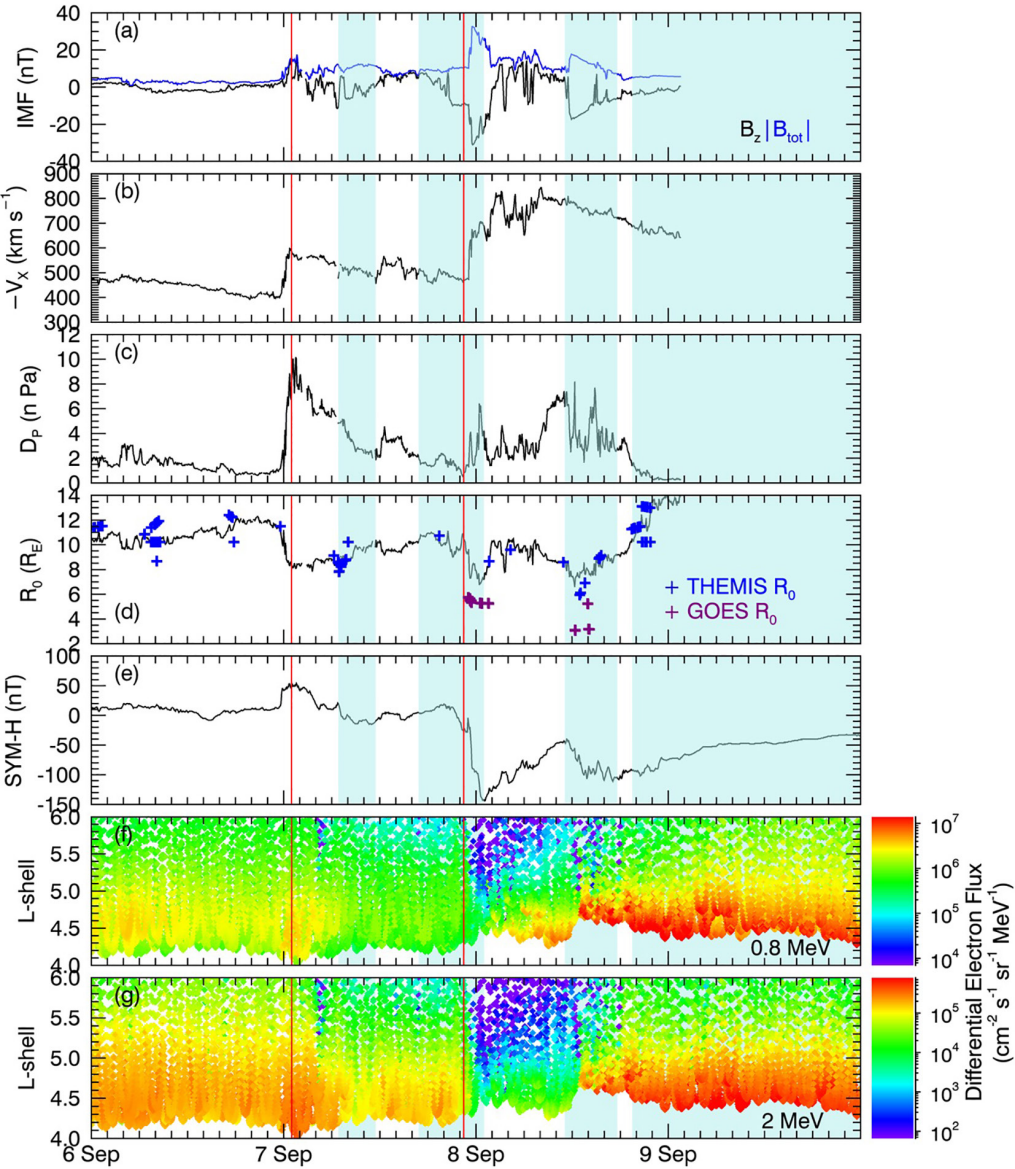
The solar wind and magnetospheric conditions for the 6–10 September 2017; (a) total interplanetary magnetic field (IMF; blue) and the north‐south component of IMF *B*_Z_ (black); (b) solar wind speed; (c) solar wind dynamic pressure; (d) subsolar standoff distance of the magnetopause, calculated by the Shue et al. (1998) model (black line), and equivalent subsolar standoff distance measured by THEMIS (blue) and GOES (purple) spacecraft according to Staples et al. (2020); (e) SYM‐H index; differential electron flux as a function of *L* shell, measured by Global Positioning System (GPS) satellites at (f) 0.8 MeV and (g) 2 MeV. Interplanetary shocks are indicated by red lines and blue shaded areas show when there are coronal mass ejecta (CME) ejecta as stated by Shen et al. (2018).

Figure 2 shows a summary of solar wind, interplanetary magnetic field (IMF), magnetospheric parameters, and radiation belt electron flux between the 6 and 10 September 2017. During the time period, there is a complex sequence of interacting interplanetary shocks and coronal mass ejecta (CME; Scolini et al., 2020; Shen et al., 2018; Werner et al., 2019) which drives an equally complex magnetospheric and radiation belt response. The times of the interplanetary shocks and CME ejecta classified by Shen et al. (2018) are displayed by the red lines and blue shaded areas, respectively. Measurements of electron flux at 0.8 and 2 MeV energies are taken by the combined X‐ray dosimeter on board LANL GPS Navstar Satellites (Tuszewski et al., 2004). As the orbits of GPS satellites are highly inclined, measurements of flux at *L* shells ≳5.5 are taken at high magnetic latitudes where fewer electrons complete their bounce orbit, and therefore flux decreases rapidly with *L* shell.

Upon the arrival of the first interplanetary shock at 01:00 UT on 7 September, there were sudden increases to solar wind speed by 200 km s^−1^, dynamic pressure by 9 nPa, and IMF field strength by 15 nT (Figures 2a–2c). These changes in the solar wind compressed the magnetopause from 11*R*_E_ to 8*R*_E_, and an increase in SYM‐H index from ∼10 to 50 nT (Figures 2d and 2e). Both 0.8 and 2 MeV flux increased by a factor of ∼2 at all *L* shells <5 (Figures 2f and 2g). When SYM‐H index returned to the prestorm value of ∼10 nT, so did electron flux at both energies.

The first CME ejecta arrived at 06:50 UT 7 September, accompanied by an IMF *B*_Z_ rotation between −10 and 9 nT, and return to −8 nT, and there is an overall decrease in solar wind velocity and dynamic pressure (Figures 2a–2c). There was little change to magnetospheric conditions with no significant change in SYM‐H, which returned to ∼0 nT prior to the first ejecta (Figure 2e), and the magnetopause was located between 8 and 10*R*_E_ (Figure 2d). Electron flux decreased by a factor of ∼4 compared to the prestorm flux, for both energies, and at all *L* shells (Figures 2f and 2g).

The second CME arrived at 16:50 UT 7 September and lasted until 01:00 UT 8 September, during which a second interplanetary shock (associated with the third CME) arrived at 22:28 UT 7 September. Upon the arrival of the interplanetary shock, IMF field strength suddenly increased from 10 to ∼35 nT and *B*_Z_ decreased further from −10 to −32 nT, the solar wind speed increased from 500 to 700 km s^−1^, and dynamic pressure increased from 1 to 4 nPa (Figures 2a–2c). The magnetopause was compressed within geostationary orbit (∼6.6*R*_E_), measured by GOES 13. The SYM‐H index decreased from 0 nT to a minimum of −142 nT, indicating the storm main phase. The 0.8 MeV electron flux decreased by a factor of ∼100 across *L* shells >5 and by a factor of ∼10 for *L* shells <5. Similarly, 2 MeV electron flux decreased by a factor of ∼1,000 for *L* shells >5 and by a factor of ∼100 at *L* shells <5. At both energies, this dropout in flux started at the highest *L* shells first, followed by the lower *L* shells over a 3‐hr period.

Following the second CME ejecta, the solar wind speed remained high at 800 km s^−1^, while dynamic pressure fluctuated, increasing from 1 to 6 nPa (Figures 2b and 2c). IMF *B*_Z_ also fluctuated rapidly between −10 and 10 nT (Figure 2a). SYM‐H began to increase (Figure 2e), indicating that the start of the storm recovery phase. The magnetopause expanded outwards to ∼10*R*_E_, as measured by THEMIS (Figure 2d). Electron flux increased across both energies; 0.8 MeV flux increased by a factor of ≳10, first limited to *L* shells <4.5 but slowly expanding to all *L* shells prior to the third CME arrival (Figure 2f). The 2 MeV electrons showed a similar increase in flux, though the rate of increase was slower (Figure 2g).

The third CME ejecta arrived on 8 September at 11:05 UT and lasted until 17:38 UT. Solar wind speed remained high between 700 and 800 km s^−1^, and solar wind pressure fluctuated rapidly between 2 and 8 nPa (Figures 2b and 2c). IMF strength suddenly increased by 8 nT, and *B*_Z_ rapidly decreased to −10 nT, slowly returning to ∼0 nT by the end of the CME (Figure 2a). SYM‐H index decreased from −50 nT to a minimum of −120 nT (Figure 2e), indicating a compound geomagnetic storm. The magnetopause was compressed within GEO orbit, as measured by GOES 13 and 15 magnetopause crossings (Figure 2f). The Shue et al. (1998) model is compressed to 7*R*_E_ at the subsolar point, and the equivalent subsolar magnetopause calculated from GOES 13 crossing was 5.5*R*_E_, and 3*R*_E_ for GOES 15. In actuality, the magnetopause is not compressed to this level at the subsolar point as Van Allen Probe A is at apogee near noon at this time and does not cross the magnetopause. This inconsistency between observations is due to errors in equivalent subsolar standoff calculation, introduced by inaccuracies in the Shue et al. (1998) model shape. Simultaneous to the compression of the magnetopause, there is a sudden increase in flux for *L* shells <5; the 0.8 MeV increased by a factor of ∼10 and 2 MeV flux increased by a factor of ∼100 (Figures 2f and 2g). At *L* shells >5, there is some reduction in flux, at both energies, compared to the flux at the beginning of the third CME.

The fourth CME ejecta arrived at 19:30 UT 8 September and continued until 00:00 UT 11 September. Solar wind conditions were no longer recorded in the 5‐min resolution OMNI‐database during this period (Figures 2a–2c). The magnetopause expanded to 13.5*R*_E_, as measured by THEMIS on 8 September (Figure 2d). For the remainder of the time period, electron flux at both energies, and all *L* shells, continued to increase to values ∼10 times greater than the prestorm flux (Figures 2f and 2g). The SYM‐H index slowly increased during the recovery phase of the storm (Figure 2e).

## 4 Phase Space Density Observations

**Figure 3:**
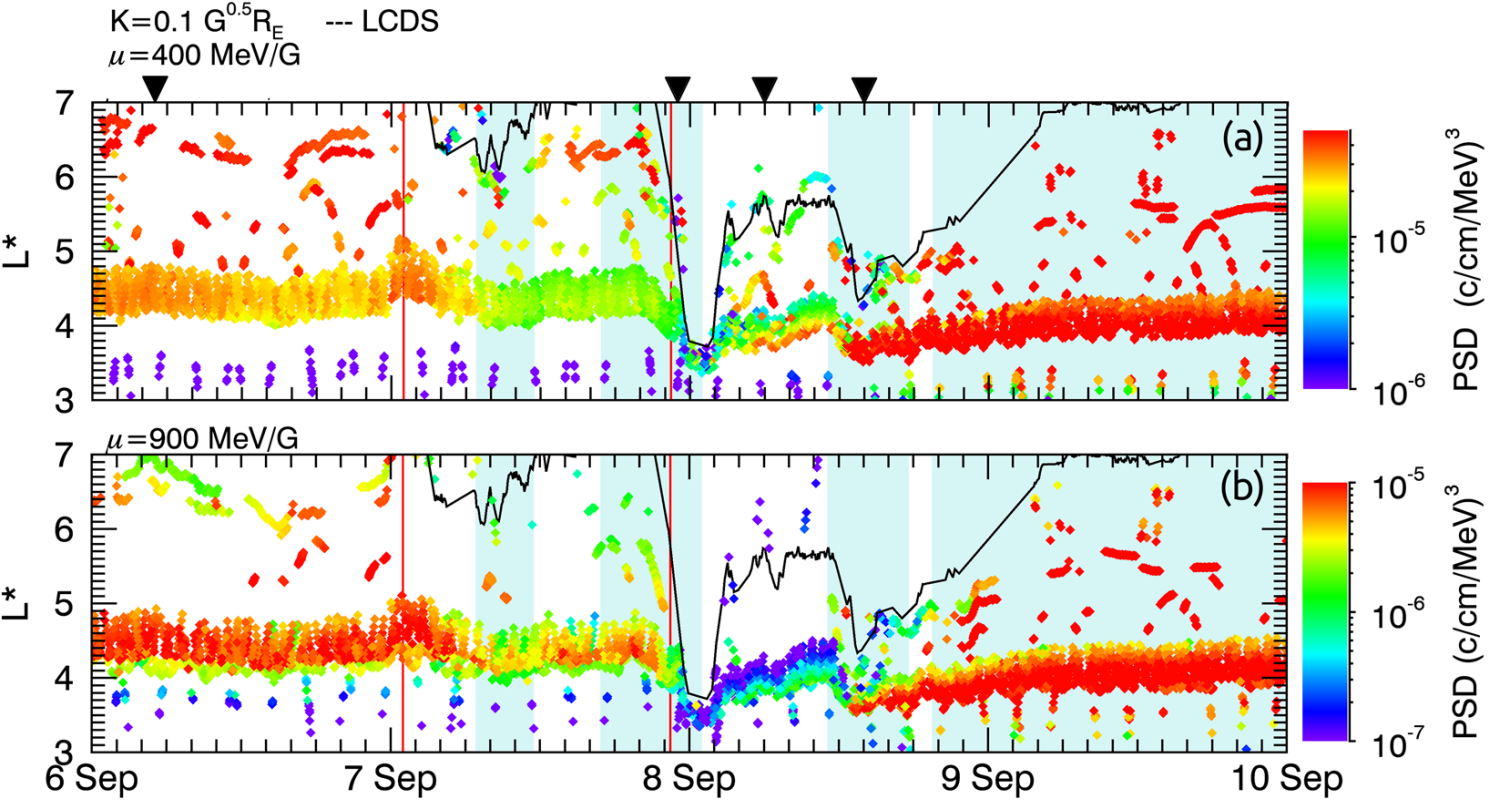
Phase space density (PSD) shown as a function of L* over time as measured by multiple spacecraft for 6–10 September 2017; panels show PSD values where *K* = 0.1 G^0.5^ *R*_E_ and *μ* is (a) 400 MeV/G; (b) 900 MeV/G. Interplanetary shocks are indicated by red lines, and blue shaded areas show when there are coronal mass ejecta (CME) ejecta as stated by Shen et al. (2018). The last closed drift shell (LCDS) for *K* = 0.1 G^0.5^ *R*_E_ is overplotted in the black solid line on all three panels. The black triangles indicate the time periods depicted in Figure 3.

Figure 3 shows PSD of electrons measured by Van Allen Probes, THEMIS, MMS, GOES, and GPS. The evolution of PSD where *K* = 0.1 G^0.5^ *R*_E_ and *μ* = 400 and 900 MeV/G (panels a and b, respectively) is shown as a function of L*. As with Figure 2, solar wind features are indicated by the red lines (interplanetary shocks) and blue shaded regions (CME ejecta). During the prestorm interval on the 6 September, PSD at *μ* = 400 MeV/G increased monotonically with increasing L*, with most electrons located at L* > 4. PSD showed that most electrons at *μ* = 900 MeV/G was also primarily at L* > 4, though PSD was peaked at L* = 4.2, and slows decreased by ∼1 order of magnitude by L* = 6.7.

The net‐loss phase in the radiation belts began prior to the main storm phase, upon the arrival of the first CME at 06:50 UT 7 September. The LCDS moved inwards to L* ∼ 6, and PSD decreased across both *μ*, though by different magnitudes depending on *μ* and L*. PSD of electrons at *μ* = 400 MeV/G halved between 4 < L* < 5 and decreased by a factor of ∼3 at L* > 5. PSD of electrons at *μ* = 900 MeV/G decreased by a factor of ∼5 between 4 < L* < 5 and decreased by a factor of ∼3 at L* > 5.

Following this initial decrease of the LCDS, the PSD remained relatively constant until the arrival of the second interplanetary shock, which coincided with the main storm phase. The magnetopause was compressed within geostationary orbit, and the LCDS reached a minimum L* of 3.8 for 2 hr. During this interval, the PSD at L* > 3.8 could therefore not be expressed in adiabatic coordinates, so we assume these particles no longer followed closed drift paths. At L* < 3.8, PSD did not change significantly for *μ* = 900 MeV/G elections, and PSD doubled for electrons at *μ* = 400 MeV/G. We note that L* sampled by the spacecraft decreased during the compression, indicating that electron drift paths moved outwards with respect to spacecraft orbits.

Following the second CME, the storm recovery phase began, and the LCDS increased to L* = 5.5. In tandem, the location of spacecraft measurements in L* moved outwards. At high L* where drift paths were previously open, PSD of electrons at *μ* = 400 MeV showed little to no change compared to before the interplanetary shock arrival, whereas PSD at *μ* = 900 MeV/G dropped out by a factor of 10. Following this, PSD increased at L* ∼ 4, by a factor of ∼10 for electrons at both *μ*.

Upon the arrival of the third CME ejecta, the LCDS was compressed a second time to L* = 4.4. Simultaneously, PSD at both *μ* increased by a factor of 10 at L* = 3.6, and PSD decreased with increasing L*. As the LCDS began to expand outwards in L*, PSD began to increase at all L*. When the fourth CME ejecta arrived, the LCDS continued to expand to higher L*, and PSD universally increased at all L* and *μ* plotted.

**Figure 4:**
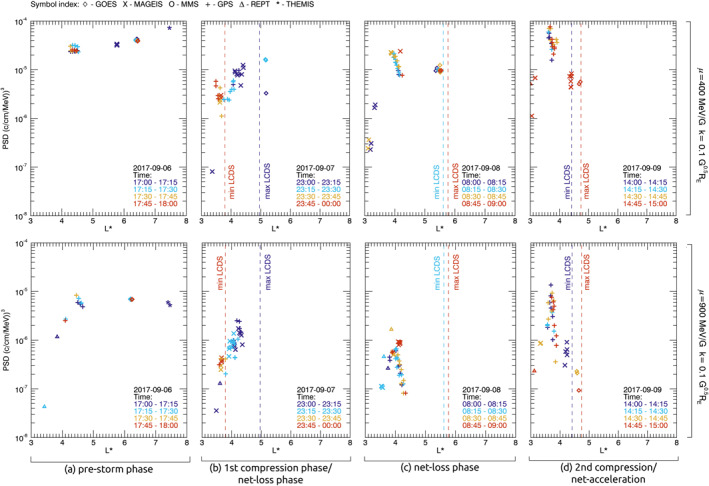
Phase space density (PSD) profiles in L* for *μ* of 400 MeV/G (top row) and 900 MeV/G (bottom row) with *K* = 0.1 G^0.5^ *R*_E_. Each column (a–d) shows a single hour time period through different phases of the storm. Symbol colors indicate when measurements were taken within the hour period. Dotted lines show the minimum and maximum L* of the last closed drift shell (LCDS).

To further evaluate the dynamics of radial evolution of PSD, Figure 4 shows PSD profiles across L* four 1‐hr time periods through the September storm. Intervals were selected by considering the phase of the storm (i.e., prestorm, net‐loss, or net‐acceleration), the data availability of PSD measurements, and whether there were coinciding magnetopause measurements to define the outer boundary. The four time periods correspond to (a) prestorm, (b) first compressive/net‐loss phase, (c) net‐loss phase, and (d) second compressive/net‐acceleration phase. Within the hour time periods, 15‐min intervals are identified by symbol color so that changes to PSD and the LCDS within the hour can be identified. To give the four 1‐hr time periods in the context of the storm as a whole, the time intervals are indicated by black triangles in Figure 3.

During the prestorm interval, Figure 4a shows that PSD of electrons at *μ* = 400 MeV/G increased monotonically as a function of L* to a maximum of 8 × 10^−5^ (c/cm/MeV)^3^ at L* = 7.4. PSD of electrons at *μ* = 900 MeV/G reached a peak of 1 × 10^−5^ (c/cm/MeV)^3^ at L* = 4.2, and PSD decreased slightly with increasing L* greater than this. During this interval, the LCDS was located at L* > 8.

During the first compression/dropout phase (Figure 4b), there were strong magnetopause compressions within geostationary orbit, and the LCDS decreased from L* = 5 to 3.8. PSD profiles showed a rapid change over the hour. Initially PSD at both *μ* values increased with L*, and PSD was measured at a maximum near the maximum LCDS for the interval. In the latter half hour, electrons beyond L* = 3.5 follow open drift paths, so will be lost through direct magnetopause shadowing. PSD measurements at L* < 3.8 showed that, while PSD of electrons at *μ* = 900 MeV/G changed little, PSD of electrons at *μ* = 400 MeV/G doubled through the latter half hour. If PSD dynamics within the hour interval were not considered, then these observations would be interpreted as false peaks or troughs in the PSD. These false peaks and troughs are produced by the dynamics of the system rather than nonadiabatic acceleration or loss processes.

Later during the net‐loss phase (Figure 4c), the magnetopause was expanded and LCDS was located at L* ∼ 5.7 throughout the interval. PSD at both *μ* peaked at L* = 3.8, and increased by a factor of 10 or more at L* < 3.8. At L* > 3.8, PSD of electrons at *μ* = 400 MeV/G PSD decreased with increasing L*, until L* = 4.2. PSD of electrons at *μ* = 900 MeV/G showed a strong negative gradient with increasing L* > 3.8, decreasing by orders of magnitude. These observations are consistent with electron loss due to direct magnetopause shadowing at L* > 3.8 (Figure 4b), followed by a redistribution of electrons via radial diffusion when the magnetopause expanded (Figure 4c).

During the secondary compression phase (Figure 4d), there magnetopause was measured within geostationary orbit, and the LCDS was located at a minimum of L* = 4.4. Both *μ* showed growing peaks in PSD at L* = 3.7. PSD at L* < 3.7 increased by a factor ≥10 compared to the previous interval (Figure 4c), and PSD at L* > 3.7 showed strong negative gradients with increasing L* for both *μ*. This is characteristic of a localized nonadiabatic acceleration process, likely due to resonant VLF wave‐particle interactions, combined with radial diffusion redistributing electrons down PSD gradients (inwards for L* < 3.7 and outwards for L* > 3.7), thus increasing PSD at any given L*. Despite the large PSD enhancements at L* < 3.7, PSD at L* > 4 remained orders of magnitude less than compared to the prestorm phase; for *μ* = 400 MeV/G, PSD was up to 10 times less than the prestorm interval, for *μ* = 900 MeV/G PSD was up to 1,000 times less than the prestorm interval. Electrons at these L* did not increase at the same rate during the early recovery phase due to ongoing losses to the magnetopause. At L* > 4.4 electrons will be lost to the magnetopause via direct magnetopause shadowing. Furthermore, strong negative gradients in PSD toward the compressed LCDS suggests that electrons transported away from the acceleration region were subsequently lost through indirect magnetopause shadowing during the early recovery phase.

**Figure 5:**
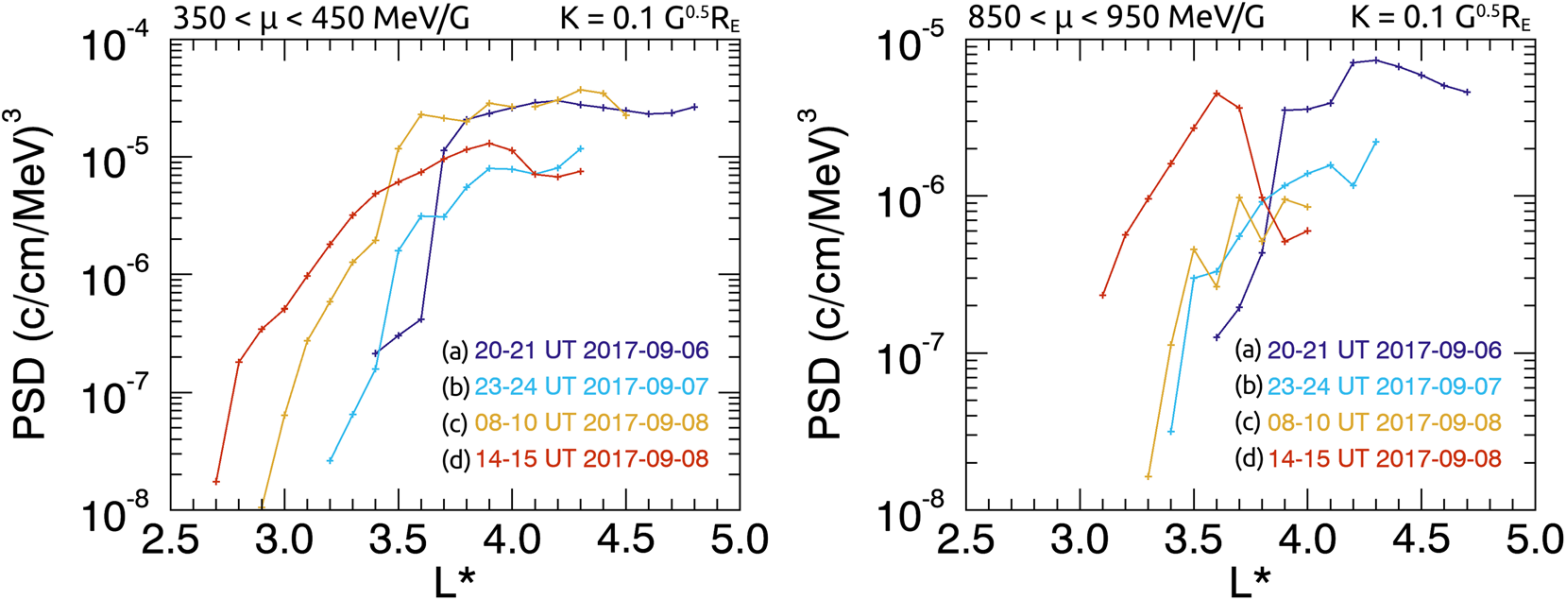
Phase space density (PSD) profiles in L* for (i) 350 < *μ* < 450 MeV/G and (ii) 850 < *μ* of 950 MeV/G with *K* = 0.1 G^0.5^ *R*_E_, as measured by the Van Allen Probes. Profiles a, b, and d show hour long time periods, c shows a 2‐hr long time period, through different phases of the storm. PSD measurements within the specified *μ* range is linearly interpolated across L* for each hour interval with a resolution of L* = 0.1.

To evaluate whether using the multisatellite data set is a beneficial addition to the Van Allen Probe data, Figure 5 shows PSD profiles in L* using Van Allen Probe data only. The time at which each profile was measured is indicated by the color, with a resolution of 1‐hr. Figure 5 intervals a–d are chosen to correspond to the intervals shown in Figure 4. In order to maximize data coverage across L*; (i) a larger range of *μ* is used in Figure 5 than was used for multimission data in Figures 3 and 4, (ii) the prestorm interval (Figure 5a) is at 20 UT on 6 September, which is 3 hr later than Figure 4a, to account for the orbital configuration of Van Allen Probes, (iii) and the net‐loss interval (Figure 5c) was extended to 2 hr at 8–10 UT on 8 September. The range of *K* is the same (0.075 < *K* < 0.125 G^0.5^ *R*_E_).

The development of PSD at 350 < *μ* < 450 MeV/G measured by the Van Allen Probes (Figure 5) showed similar features between net‐loss intervals (Figures 5a–5c) to observations of PSD at *μ* = 400 MeV/G using the multimission data (Figure 4). During the final interval (Figure 5d), the PSD peak at L* = 3.7 observed by multimission data (Figure 4d) was not observed since there were no Van Allen observations between 3.2 < L* < 4.1 at the specified range of *μ* (350–450 MeV/G).

The development of PSD at 850 < *μ* < 950 MeV/G measured by the Van Allen Probes (Figure 5) showed similar features between intervals Figures 5a, 5b, and 5d, to observations of PSD at *μ* = 900 MeV/G using multimission data (Figures 4a, 4b, and 4d). During the net‐loss phase (Figure 5c), the Van Allen Probes did not measure the negative PSD gradient at L* > 3.8 observed by multimission data (Figure 4c) since there were no Van Allen observations at L* > 4 at the specified range of *μ* (850–950 MeV/G).

## 5 Discussion

We presented an overview of the outer electron radiation belt response to a sequence of interacting CMEs and interplanetary shocks propagating through the solar wind in early September 2017. We used multimission, multispacecraft observations of both electron flux and phase space density to evaluate the role of magnetopause shadowing in producing changes to electron flux during this event.

Between 7 and 10 September, there were two interplanetary shocks and four separate CME ejecta propagating through the solar wind. As the solar wind characteristics of this event have already been studied in detail (e.g., Scolini et al., 2020; Shen et al., 2018; Werner et al., 2019), we do not analyze these in detail, instead we focus on the relativistic electron response to these drivers. There was a complex response in electron flux at 0.8 MeV and/or 2.0 MeV energies corresponding to each interplanetary shock or CME ejecta. Most of these flux changes corresponded to changes in measurements of PSD, indicating that the electron flux response to solar wind driving were largely due to nonadiabatic processes.

Not all changes in electron flux were due do nonadiabatic processes. Specifically, in response to the second interplanetary shock arrival, flux measured by GPS satellites decreased by orders of magnitude at all *L* shells. Corresponding PSD measurements showed that, while electrons were irreversibly lost at high L* where drift paths were intersected by the LCDS, there were initially either increases, or no changes, to PSD measured below L* = 3.8. During this interval, the location in L* at which GPS satellites was measuring PSD changed, i.e., electron drift paths had moved radially outwards with respect to GPS orbits. Therefore, GPS satellites measured a different part of the PSD distribution, which appeared as a decrease in the measured electron flux on all *L* shells. This shows why it is important to consider electron measurements in adiabatic coordinates, rather than flux alone.

Observations of PSD, and motion of the magnetopause, showed that magnetopause shadowing was the dominant loss mechanism responsible for an electron flux dropout during the main storm phase. As the magnetopause became compressed, the LCDS decreased to L* = 3.8 for 2 hr. Electrons beyond this L* followed drift paths which intersected the magnetopause, and were assumed to be lost via “direct” magnetopause shadowing on the time scale of an electron drift orbit (Figure 4b). As the magnetosphere expanded, the PSD at L* where drift paths were previously open showed electron losses by orders of magnitude. This resulted in a PSD distribution which was peaked at L* = 3.8 (Figure 4c).

Following the dropout of electrons, a growing peak in PSD was observed at L* = 3.7 (Figure 4d). This peak in PSD cannot be explained by magnetopause shadowing, so was attributed to a localized acceleration mechanism which enhanced PSD by orders of magnitude. It was further concluded that radial diffusion acted to transport electrons down the gradients created in PSD, since PSD at L* < 3.7 increased in conjunction with the growing peak. This is supported by Van Allen Probe observations of ULF wave, which show enhanced ULF wave activity through the storm (Figure S1 in Supporting Information S1).

Despite strong evidence that radial diffusion acted to transport electrons away from the acceleration region, PSD at L* > 4.4 did not increase in conjunction (Figure 4d). This was attributed to “indirect” magnetopause shadowing, where electrons diffused to high L* were transported toward the compressed magnetopause and subsequently lost. As a result, the negative PSD gradient created by earlier magnetopause shadowing was maintained, and PSD enhancements were limited to low L* at the beginning of the acceleration phase (14–15 UT 8 September).

The observations of PSD presented in this study are consistent with the framework of magnetopause shadowing discussed in previous works (e.g., Loto'aniu et al., 2010; Shprits et al., 2006; Turner et al., 2012), but were shown to significantly contribute to the overall dynamics of PSD during the net‐acceleration phase. Unfortunately, without knowing the number of particles accelerated during the local acceleration, we cannot measure the number of particles lost via indirect shadowing to evaluate the respective contributions of direct and indirect shadowing to overall electron loss. In future work, the contributions of each mechanism could instead be estimated by replicating this event with a radiation belt model which includes both source and loss mechanisms.

We highlight that the time and spatial resolution of the multimission PSD data set enhances observations and improves our understanding of the complex spatiotemporal dynamics of the outer radiation belt. Figure 5 showed observations of the PSD profiles as a function of L* corresponding to intervals shown in Figure 4, using only Van Allen Probe observations. Through the intervals shown, the maximum L* measured by the Van Allen Probes was L* = 4.8, whereas the multimission data measured up to L* = 7.5. Though this difference is less important during the compressive phases, large L* regions of PSD are not measured by the Van Allen Probes during the prestorm and recovery phases. Furthermore, key observations necessary to accurately characterize the development PSD through the storm are not present when only analyzing Van Allen Probes data. Namely, the growing PSD peak at L* = 3.7 was not observed for *μ* ∼ 400 MeV/G, and the negative PSD gradient at L* > 3.7 resulting from direct magnetopause shadowing (Figure 4c) was not observed in Van Allen Probe observations at *μ* ∼ 900 MeV/G. Olifer et al. (2021) also observed that the PSD measured by the Van Allen Probes could produce misleading results during the September 2017 storm, when “phantom peaks” where observed. However, the high‐resolution multimission data set used in our analysis resolved that the observed peak in PSD was indeed growing on very fast time scales for electrons with *μ* of 400 and 900 MeV/G, which we attribute to local acceleration. It is clear that, in order to understand radiation belt dynamics, measurements of PSD on time scales shorter than the Van Allen Probe orbits are crucial.

Finally, we acknowledge possible limitations to this work, specifically the magnetic field model used as it is known to influence calculations of the adiabatic invariants and LCDS (e.g., Albert et al., 2018). The T01s model was chosen for this work due to its suitability during geomagnetic storm conditions, but different magnetic field models may yield a different L* location of the LCDS. We recommend that future work should assess the influence of different magnetic field models on PSD computations in adiabatic coordinates. Furthermore, the LCDS may be calculated by either considering Shabansky drift orbits as closed drift paths, whereas other calculations consider these bifurcating orbits as open (Shabansky, 1971; Öztürk & Wolf, 2007). In this work, we have used the last nonbifurcated drift shell as the LCDS, which could give an underestimation of the outer boundary to trapped electrons if these electrons following bifurcated orbits return to the radiation belts following the compression. Despite this, these limitations are unlikely to change the interpretation of our observations as PSD profiles show clear evidence of magnetopause shadowing, outward radial transport, and local acceleration.

## 6 Summary and Conclusions

We used multipoint electron phase space density measurements from 32 satellites which provided unprecedented temporal and spatial resolution to analyze magnetopause shadowing in the outer radiation belt during the early September 2017 geomagnetic storm. The September 2017 storm is driven by a set of complex solar wind features, due to four interacting CMEs, with an equally complex radiation belt response.

Analysis of multispacecraft electron flux and PSD measurements showed capability to identify and isolate intervals of loss, energization, and mixed intervals of acceleration at low L* and loss at high L*:

1. By comparing PSD to flux measurements, we identified adiabatic transport of electrons during the event, highlighting the importance of considering adiabatic coordinates to interpret flux changes during geomagnetic storms
2. An electron flux dropout was shown to be predominantly produced by “direct” magnetopause shadowing, which occurred during strong magnetopause compressions within geostationary orbit
3. Magnetopause shadowing continued to influence radiation belt dynamics during the recovery phase of the storm, in conjunction with acceleration processes at low L*

Finally, we demonstrated that Van Allen Probe data alone was not sufficient to correctly interpret the fast acceleration and loss processes which were identifiable with the extremely high spatial and temporal resolution of multispacecraft measurements. This emphasizes the need to expand analysis beyond dual spacecraft observations of PSD which cannot always capture the rapid time scales of these complex dynamics.

## Supporting information

Supporting Information S1

## Data Availability

Multimission phase space density observations presented in this study are publicly available via https://doi.org/10.5281/zenodo.5639076. We gratefully acknowledge the CXD team at Los Alamos National Laboratory for GPS data, which may be accessed via https://www.ngdc.noaa.gov/stp/space-weather/satellite-data/satellite-systems/gps/. Solar Wind data and geomagnetic indices are publicly available through the NASA/GSFC Space Physics Data Facility OMNIWeb service (https://omniweb.gsfc.nasa.gov/).
