# Supplementary material for: Resolving Magnetopause Shadowing Using Multimission Measurements of Phase Space Density
Source: J Geophys Res Space Phys. 2022 Jan 27;127(2):e2021JA029298. doi: 10.1029/2021JA029298 (PMC9286781; doi:10.1029/2021JA029298)
Supplement: Supplementary file 1 — Supporting Information S1 [file JGRA-127-0-s001.docx]

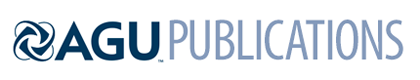


*JGR: Space Physics*

Supporting Information for

**Resolving Magnetopause Shadowing Using Multi-Mission Measurements of Phase Space Density**

F.A Staples^1^, A. Kellerman^2^, K. R. Murphy^3^, I. J. Rae^3^, J. K. Sandhu^3^, C. Forsyth^1^

^1^Mullard Space Science Laboratory, University College London, London, UK.

^2^Department of Earth, Planetary, and Space Sciences, University of California, Los Angeles, USA.

^3^Northumbria University, Newcastle upon Tyne, UK.

**Contents of this file**

Figure S1

**Introduction**

To understand how radial diffusion will have acted upon the electron populations shown during the September 2017 geomagnetic storm, we use Van Allen Probe electric and magnetic field measurements to analyze the power of field fluctuations. We used in-situ magnetic field observations from the Van Allen probe EMFISIS instrument (Kletzing et al., 2013) and electric field observations from the EFW instruments (Wygant et al., 2014). The magnetic and electric field measurements had a time resolution of 4 seconds. The background magnetic and electric fields were identified by taking a running average over a 20-minute sliding window, and the background fields were then subtracted from the instantaneous measurement. To identify fluctuations, the residual field observations were transformed into a magnetic field-aligned coordinate system, defined by the background magnetic field unit vector (parallel component), the geocentric position vector (azimuthal component), and the poloidal direction. In this transformed coordinate system, the parallel magnetic field perturbation and azimuthal electric field perturbation were selected to estimate the associated power spectral densities using a Morlet wavelet transform. The power spectral density was limited to a frequency range of 1-15 mHz (corresponding to the ULF wave band (Jacobs et al., 1964)) and an L-range of 3 < L < 7.5 as field perturbations cannot be reliably distinguished from the changing background field near orbital perigee.

Figure S1 shows the power spectral density of the magnetic field P_B_ (green) and electric field P_E_ (pink) as a function of L* for the 4 previously specified 1-hour intervals (Figure 4). Magnetic power spectral density is substantially enhanced at an increasing rate through the geomagnetic storm (Figure S1, panels b - d) compared to the pre-storm interval (Figure S1a). Due to data gaps in electric field measurements (associated with spacecraft charging and eclipse events), the number of electric field power spectral density measurements is variable throughout the storm, making it difficult to make comparisons between the four intervals (e.g., Figure5a&d). However, during the first compressive phase (Figure S1b), P_E_ appears high at low L*, and decreases with increasing L*. During the net-loss phase (Figure S1c), P_E_ decreased at all L* compared to the previous compressive phase. During the second compressive phase (Figure S1d), there was only one measurement of P_E_ at L*=3.7, which was increased compared to the previous net loss phase (Figure S1c). Auroral geomagnetic indices (not shown) indicate enhanced substorm activity during this interval, which will produce fluctuations in electric potential in the magnetosphere, and thus contribute towards the power spectral density of the electric field. It is therefore highly likely that P_E_ was enhanced during interval (d) in a similar manner to interval (b).

**References**

Jacobs, J., Kato, Y., Matsushita, S., & Troitskaya, V. (1964). Classification of geomagnetic 747 micropulsations. Journal of Geophysical Research, 69(1), 180–181. doi: 748 https://doi.org/10.1029/JZ069i001p00180

Kletzing, C., Kurth, W., Acuna, M., MacDowall, R., Torbert, R., Averkamp, T., Bodet, D., Bounds, S., Chutter, M., & Connerney, J. (2013). The electric and magnetic field instrument suite and integrated science (EMFISIS) on RBSP*. Space Science Reviews*, 179(1-4), 127-181. https://doi.org/10.1007/s11214-013-9993-6

Wygant, J. R., Bonnell, J. W., Goetz, K., Ergun, R. E., Mozer, F. S., Bale, S. D., … Tao, J. B. (2014). The Electric Field and Waves Instruments on the Radiation Belt Storm Probes Mission. In N. Fox & J. L. Burch (Eds.), The Van Allen Probes Mission (pp. 183-220). Springer US. https://doi.org/10.1007/978-1-4899-7433-4_6


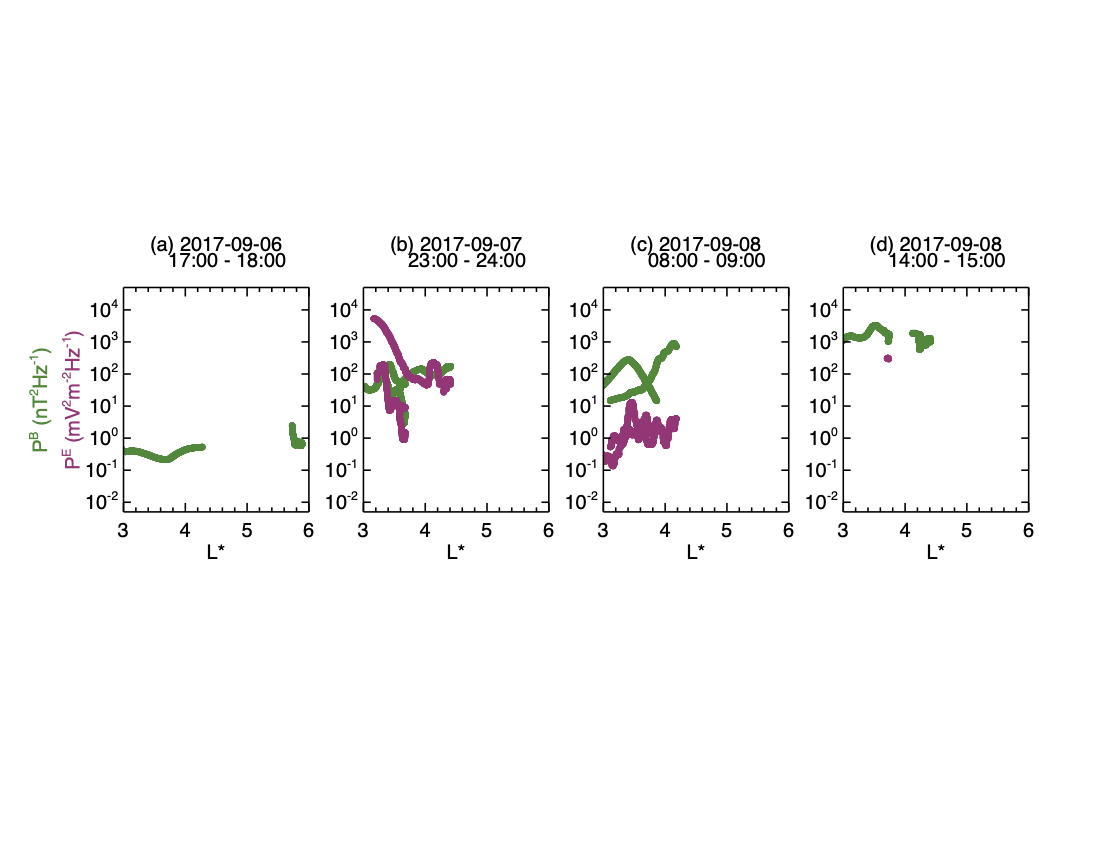


Figure S1. Estimated magnetic field (green) and electric field (purple) power spectral densities for (a) 17 - 18 UT 6 Sept, (b) 23 UT 7 Sept to 00 UT 8 Sept, (c) 08 – 09 UT 8 Sept, and (d) 14 - 15 UT 8 Sept. Each panel shows the power spectral density, summed over a frequency range of 1 to 15 mHz, plotted as a function of the L* value. Observations from both Probe A and Probe B are included.
